# Hardware Efficient Direct Policy Imitation Learning for Robotic Navigation in Resource-Constrained Settings

**DOI:** 10.3390/s24010185

**Published:** 2023-12-28

**Authors:** Vidura Sumanasena, Heshan Fernando, Daswin De Silva, Beniel Thileepan, Amila Pasan, Jayathu Samarawickrama, Evgeny Osipov, Damminda Alahakoon

**Affiliations:** 1 Research Centre for Data Analytics and Cognition, La Trobe University, Bundoora, VIC 3083, Australia; 2 Department of Computer Engineering, Rensselaer Polytechnic Institute, New York, NY 12180, USA; 3 Department of Computer Science, University of Warwick, Coventry CV4 7AL, UK; beniel.thileepan@warwick.ac.uk; 4 Centre for Wireless Communications, University of Oulu, 90570 Oulu, Finland; 5 Department of Electronic and Telecom Engineering, University of Moratuwa, Moratuwa 10400, Sri Lanka; 6 Department of Computer Science, Electrical and Space Engineering, Luleå University of Technology, 97187 Luleå, Sweden

**Keywords:** imitation learning, direct policy learning, autonomous navigation, mobile robots

## Abstract

Direct policy learning (DPL) is a widely used approach in imitation learning for time-efficient and effective convergence when training mobile robots. However, using DPL in real-world applications is not sufficiently explored due to the inherent challenges of mobilizing direct human expertise and the difficulty of measuring comparative performance. Furthermore, autonomous systems are often resource-constrained, thereby limiting the potential application and implementation of highly effective deep learning models. In this work, we present a lightweight DPL-based approach to train mobile robots in navigational tasks. We integrated a safety policy alongside the navigational policy to safeguard the robot and the environment. The approach was evaluated in simulations and real-world settings and compared with recent work in this space. The results of these experiments and the efficient transfer from simulations to real-world settings demonstrate that our approach has improved performance compared to its hardware-intensive counterparts. We show that using the proposed methodology, the training agent achieves closer performance to the expert within the first 15 training iterations in simulation and real-world settings.

## 1. Introduction

Reinforcement learning and imitation learning are the two primary forms of machine learning used currently in robotics [1,2,3]. Although reinforcement learning has proven to be more effective in mobile robotics applications, building a reinforcement learning model for a real-world robot is practically challenging due to the following reasons: formulating a suitable reward function for complex manipulations, the need to compromise the safety of the robot when following a classical reinforcement learning approach, and the increasing time to learn when the state space of the problem increases. Imitation learning, or learning from demonstration (LfD), has a different approach to achieving the necessary skill needed by the robot that also overcomes these challenges [4]. An imitation learning model that is implemented in a robot is typically referred to as an agent. Here, the agent is allowed to observe, by some sensory means, an expert performing the task that the robot is ultimately intended to perform. This approach allows the learning time of the agent, for a given task, to be drastically reduced in comparison with a reinforcement learning task. This is due to the fact that general imitation learning approaches do not require extensive exploration as required in reinforcement learning. One of the main paradigms of imitation learning is direct policy learning, which directly learns the optimal behavior required by querying the expert [5]. This approach overcomes the issue of a lack of information in the expert dataset for certain states, as it has real-time access to the expert. Accessing an expert in this manner is facilitated in a simulation, as many pre-trained models are readily available. However, in a real-world setting, this could be costly. As highlighted in [6], the majority of direct policy learning (DPL) approaches have only been implemented in simulations.

In this article, we investigate the expansion of DPL into real-world environments through a lightweight and fast-converging direct policy imitation learning approach for autonomous robotic navigation in resource-constrained settings. The proposed approach is lightweight due to its low memory consumption and fast inference capabilities, which makes it suitable for resource-constrained mobile robotic environments to facilitate onboard training and decision-making. Fast convergence is realized, achieving satisfactory performance with minimum training iterations. In this context, a mobile robot is an autonomous robot vehicle capable of moving in the environment. We evaluate these capabilities through the real-world implementation of the proposed approach on a custom-designed small-scale robot vehicle equipped with navigational sensors. The results of these experiments confirm its validity and capability in autonomous navigation in resource-constrained settings.

We summarize our key contributions as follows.

1. A novel two-policy imitation learning system for navigation and safety capable of training and operating simultaneously.
2. Recent DPL systems applied to real-world tasks, such as [7,8], have complex policy models and are evaluated on powerful computing resources. Therefore, as our second contribution, we make our system lightweight with only  2000 online trainable parameters.
3. We present a comprehensive comparison of the hardware-intensive counterparts of feature extraction and policy networks.
4. We demonstrate the capabilities in simulation and extend the experiments to the real world.
5. We evaluate the system in the real world on the navigation task of traveling on a two-lane road, overtaking obstacles by partially entering the other lane, and returning to the correct lane while having the capability of braking if the obstacle is unavoidable.

The rest of the paper is organized as follows. Section 2 presents related work on vision-based autonomous systems and imitation learning, followed by Section 3, which delineates the overall composition of the proposed imitation learning approach. In Section 4, we present the configuration of the simulation and the real-world implementation of the approach. Section 5 presents the experiments and results of their functionality compared to their counterparts. Section 6 presents a discussion and concludes the paper.

## 2. Related Work

Vision-based autonomous systems are categorized broadly into two main paradigms, namely (1) mediated perception approaches and (2) behavior reflex approaches. Mediated perception [9] identifies the task of driving as a combination of several visual elements, such as lanes, vehicles, pedestrians, traffic lights, etc. with an AI-based decision maker [10]. The recognition of these visual elements is combined to generate a world representation so that the AI-based decision-maker can generate an informed steering decision. These systems are modular and usually decouple the path planning and control from the initial perception step [11]. Most autonomous driving systems from the industry today are based on mediated perception approaches [12]. Our work falls under the mediated perception paradigm, as we divide the perception and control decision into two separate modules. Related works include the detection of vehicles and output bounding boxes [13,14]. Identifying lane markings in urban driving settings and highways is addressed in [15,16,17,18,19].

Behavior reflex methods try to construct a direct map between the sensory input and driving action. For behavior reflex approaches, early works such as [20,21] laid the foundation to use a neural network to map images directly to steering control. More recent work [22] has proposed a 3-camera system that trains end-to-end via imitation learning. In Ref. [23], the authors tried to improve the explainability of these end-to-end black boxes by training an imitation learning agent with an attention model.

Similarly, deep learning has its own approaches to robotic vision. Most work involves object detection (pedestrians, vehicles, etc.), scene representation, spatiotemporal vision, visual place recognition, semantic segmentation, and scene depth estimation. Since our work is based on scene representation, we focus special attention on it. Classical approaches for representing scenes are based on hand-crafted features. Deep learning has solved a variety of related tasks ranging from encoding scene information to performing navigation [24].

In Ref. [25], the authors used deep convolutional neural network (CNN) receptive field-based deep features to perform image recognition. In Ref. [26], a CNN-based appearance-invariant image encoding system was proposed. Each image was encoded to a 128-dimensional vector such that images of the same location under severe weather changes have a lesser Euclidean distance.

In Ref. [27], intermediate layers of a pre-trained CNN were used as image descriptors, outperforming state-of-the-art SLAM loop closure detection algorithms under significant lighting changes. In Ref. [28], the authors proposed a method to overcome the well-known closed-set limitation in computer vision with a CNN coupled with one-vs.-all classifiers. Domain knowledge is included by embedding a Bayesian filter. The final output is a semantic map representing the scene that can be used to boost object detection and enhance behavior during navigation tasks. In Ref. [29], scene representation is performed by incorporating object-level information using CNNs. However, most of these deep learning approaches suffer from high resource consumption in terms of processing power and memory [24]. Ref. [24] showed that the speed of vision-based pipelines for robotic applications is limited by the CNN model. GoogLeNetl a 22-layer deep CNN [30], used 119.0 W for 138 images/second on a GTX Titan X and 4.0 W for 33 images/second on a GTX Tegra X1, which are GPUs manufactured by NVIDIA Corporation, Santa Clara, CA, during 1-batch-size inferencing [31]. Titan X is a resourceful GPU equipped with 3072 CUDA cores and 12 GB of memory, while Tegra X1 has 256 CUDA cores with 4 GB of memory, thus explaining this clear performance difference. In our work presented here, we try to overcome this challenge by using a lightweight CNN architecture so that it can easily fit into a resource-constrained robot environment.

Imitation learning utilizes datasets of expert demonstration (typically human) to train a system to imitate a given expert for a range of scenarios presented [6]. Imitation learning is broadly categorized into 3 main sections, namely (1) behavioral cloning, (2) inverse reinforcement learning (IRL), and (3) direct policy learning. Behavioral cloning is the most explored area in imitation learning. Behavioral cloning is a supervised learning approach that uses expert demonstrations to train an agent in an offline manner. It further can be subdivided into end-to-end control prediction, direct perception, and uncertainty quantification [6]. End-to-end control prediction tries to directly map sensory inputs to control signals similarly to behavior reflex approaches. Some works include [22,23,32,33]. IRL is the problem of inferring the hidden preferences of another agent from its observed behavior, thereby avoiding a manual specification of its reward function compared to reinforcement learning [34,35]. Some works include feature-based IRL [36,37], entropy-based IRL [38,39], and generative adversarial imitation learning (GAIL), a model-free form of the IRL algorithm [40].

Direct policy learning (DPL) builds on behavioral cloning by attempting to leverage a given expert at training time to overcome some of the limitations of behavioral cloning [6]. Our imitation learning approach is a  DPL method; therefore, here, we explore the related work in detail. One of the prominent works [41] proposed the DAgger algorithm, which is an online imitation learning algorithm. The idea was to start with a random policy for the imitation learning agent and let it engage with the environment. During the first interaction, for each state, the agent queries an expert for its control parameters for each of the states in the trajectory. This gathered dataset is used to train the initial policy of the agent. During the next episode, the agent is allowed to interact with the environment again to gather more data points for the dataset. These collected state–action pairs are aggregated to the previously collected dataset, and the policy is trained again with this new dataset. This process is repeated to gather more data points to enrich the dataset with the relevant state–action pairs for the task. In DAgger, the way the agent interacts with the environment is controlled by a proportion of expert control and the training agent so that the agent trajectories are controlled in such a way that they are not idling to unsafe or irrelevant states and they are not strictly following expert trajectories only. In initial training iterations, the expert has more control over the agent, and as the training progresses and the training agent becomes better at the task, some portion of the control is transferred to the training agent to allow for a controlled state exploration. Building on the DAgger algorithm, [42] proposed SafeDAgger to minimize the number of queries from the expert, as this is costly. It introduces an additional safety policy to predict the likelihood of the agent deviating from the expert trajectory. This improved the learning rate and reduced the number of expert queries. In both of these works, the performance is evaluated via simulations. In Ref. [43], a novel algorithm is proposed to train end-to-end systems called observational imitation learning (OIL). OIL is an online learning method that tries to imitate the best behavior out of a set of sub-optimal teachers. The Sim4CV [44] simulation environment was used to evaluate OIL.

In most of the work on DPL, implementation is carried out in simulations due to the easy access to an expert and the difficulty of deriving a reliable performance metric. In our work, we address this gap in the research by extending our DPL-based approach from simulations to the real world and propose an evaluation metric to be used in complex real-world tasks.

## 3. Methodology

**Figure 1:**
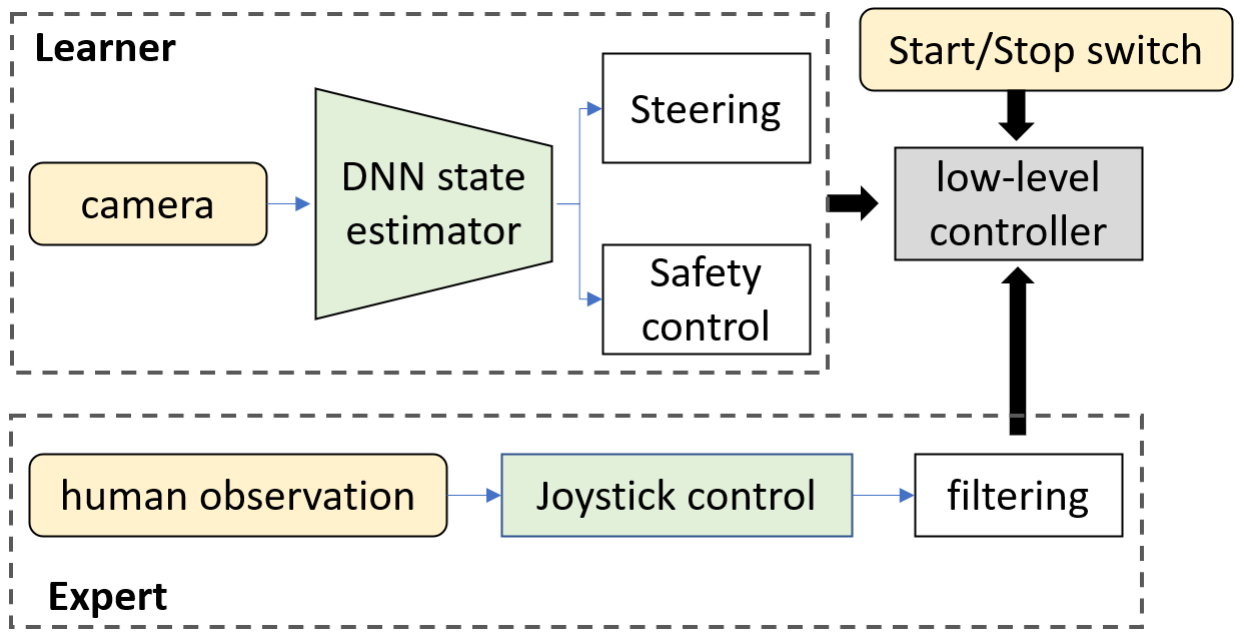
The imitation learning system. The learner feeds real-time camera input to the pre-trained DNN for state estimation. The estimated state is shared between the steering and the safety control policies for training. A start/stop switch is added to the joystick to turn on/off power to the motor driver immediately to stop the wheels in an emergency.

We propose a learning approach for autonomous navigation tasks, which takes less time in training to perform adequately well and is also lightweight enough to be capable of onboard learning. We evaluate it on a resource-constrained real-world autonomous small-scale robot vehicle. A brief overview of the approach is as follows. Overall, the system contains three main parts: the environment, the learning agent, and the expert (see Figure 1). The environment is the real or the simulated world the agent interacts with. The agent has sensors to sense this environment and actuators to interact with or alter its state in the environment. It sees the environment via its sensors, which in our work is an R GB Camera. The actuators to change its position are the motors or the wheels of thesmall-scale robot vehicle. The expert is an entity that provides the agent with knowledge of the specific task at hand in the environment. For direct policy learning, we use teleoperation in order to demonstrate the expert actions to the agent using a joystick controlled by a human. Although there are works on multi-sensor fusion for autonomous robots [45,46], we use only an R GB Camera as the sensory input mainly due to the focus in our work on the imitation learning approach. The learning system takes a front-facing R GB camera feed as the input and gives out the control output (steer angle/brake). We follow a modular pipeline consisting of the convolutional layer part, consisting of non-trainable bottom layers of MoblieNet [47], followed by a linear regressor of 1024 parameters (without bias) with tanh activation.

The advantages of the proposed method compared to the existing DPL methodologies are notable in a few aspects. One is the capability of online training. Since there are only a few thousand trainable parameters, our pipeline allows quick training compared to training policies with a few million parameters. This is advantageous especially when the expert in the Imitation learning framework is expensive. This also allows seamless implementation in real-world tasks where a human acts as an expert. The human expert does not have to wait long until the training is finished after each training episode. Another aspect is that, due to this smaller number of trainable parameters, robot vehicles do not have to be equipped with powerful hardware to facilitate real-time training. This allows the implementation of the proposed approach in resource-constrained robotic applications.

The imitation learning pipeline consists of two main components, namely the perception module and the learning module. The R GB camera inputs data to the perception module and generates a hypervector, which is a representation of the scene. A hypervector is a vector that has a higher dimensionality, usually in the order of thousands. This hypervector is the input to the learning module, which is a neural network that outputs a steering parameter. This end-to-end pipeline is depicted in Figure 1. Both the learning and perception modules will be discussed with further details in Section 3.1.

Although the environment is common to both the expert and the agent, the interpretation of the environment by each may be different or the same, depending on the type of expert. For example, a human expert has a different view of the environment than an autopilot in a simulated environment. In either case, the agent should be capable of understanding the instructions of the expert to train for a specific task. This is a known challenge in direct policy learning to expand its applications to the real world beyond simulation software [6]. In our work, we explore the capability of applying a state-of-the-art DPL in several real-world tasks.

### 3.1. Imitation Learning Approach

The imitation learning agent has two main components: the perception module and the learning module. The camera captures an instant or a state in the environment and feeds into the perception module. This module contains a pre-trained convolutional neural network (CNN) that extracts features and generates a high-dimensional representational hypervector, which becomes the input to the learning module. The learning module is a neural network (NN) that trains on the control signals of the expert that correspond to each state. The following section describes each of the modules in detail.

#### 3.1.1. Perception Module

**Figure 2:**
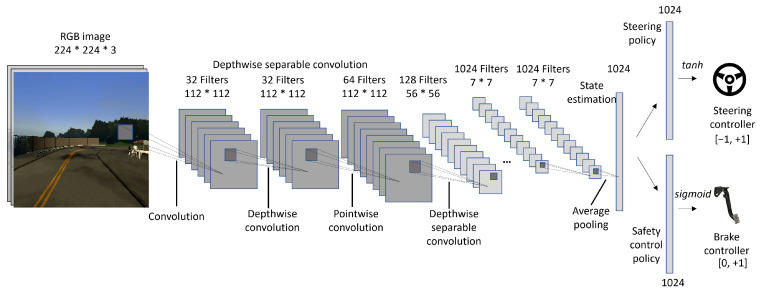
Deep CNN architecture used in the system. The state estimation vector is a 1024-dimensional vector that is shared among the steering policy and safety control policy.

For the perception module, we use the convolutional filter layers of the MobileNet [47] (excluding the fully connected layers at last), which is pre-trained on the ImageNet [48] dataset. After initializing the CNN with the pre-trained weights, we carry out transfer learning using  5000 images from a simulated driving environment by freezing all the layers except the last two. The images used for transfer learning were generated using the CARLA simulation environment version 0.9.13. [49]. We used a scene camera available in CARLA fixed on the roof of an autopilot simulation vehicle to generate these 5000 R GB images. The images were generated on different environmental conditions consisting of different times of the day and weather conditions, and for each condition of the environment, a sample of 500 images were collected. During training, we randomly dropped neurons in the last layers with a rate of 0.5 to prevent over-fitting. Then, the CNN was connected to the learning networks for steering and safety control, as shown in Figure 2. The remaining convolutional layers are not subjected to any change during the learning or driving phases of the agent. MobileNet is a lightweight convolutional neural network that uses depthwise convolution. This allows MobileNet to have a reduced number of parameters with increased computational efficiency, making it a suitable candidate for resource-limited robotics environments.

#### 3.1.2. Learning Module

The learning module is the only trainable component in the imitation learning agent. There are two neural networks, namely the steering policy and the safety control policy. The steering policy is responsible for learning the angle of steer in the range [−1,+1], where −1 means a full left turn and +1 is  a full right turn. The Safety Control Policy is responsible for controlling the throttle value which is in the range [0,1]. We also call this the braking network. For both networks, we use a linear regressor with 1024 randomly initialized weights with a bias parameter. This choice is due to two main factors. During initial experiments, we observed that the larger the learning network, the more demonstrations it needed to attain relatively good performance. With fewer trainable parameters in the network, performance converges faster to the desired task at the cost of the task being simpler. Our simulation and the real-world experiments showed that tasks such as driving in a lane and avoiding obstacles can be quickly learned with this type of simpler network. Another reason for the lightweight learning module is to limit the time and energy needed to train the agent in an online setting. In a real-world application, longer training times per episode increase the overall waiting time of the expert, which is costly. This lightweight learning module reduces this issue, which is one of the main challenges in DPL methods.

### 3.2. Learning Algorithm

The imitation learning algorithm used to train the agent is a variation of the general DAgger algorithm [41]. A human will develop a basic understanding from available information at first and incrementally refine their understanding as new information arrives [50]. Similar to this, the DAgger algorithm incrementally aggregates data points to enrich its dataset. We observed that, despite the fact that the original DAgger algorithm suggests taking the best policy after validation out of all the policies obtained after each training iteration, if the agent is allowed to learn for a sufficient time, the policies generated towards the latter part of the training episodes give the best performance. Algorithm 1 shows our proposed algorithm used in our simulations and real-world applications to train the agent.
**Algorithm 1** DAgger algorithm used in the simulation.  1:Initialize dataset D←∅  2:Initialize $\pi_{1}$ for any policy in the possible policy space  3:Define $\beta_{0}\in \left(0,1\right)$  4:**for** i=1 to *N* episodes **do**  5:      $\beta =\beta_{0}^{i-1}$  6:      Let $\pi_{i}=\beta \pi^{*}+\left(1-\beta \right)\pi_{i}$  7:      Sample *f* frames, $\left\{s_{j}\right\}$, using $\pi_{i}$  8:      $D_{i}=\left\{\left(s_{j},\pi^{*}\left(s_{j}\right)\right)\right\}$  9:      $D\leftarrow D\cup D_{i}$10:    Train policy $\pi_{i+1}$ on *D*11:**end for**12:**return** $\pi_{N}$

## 4. Setup

The proposed learning system was tested in a simulation and a real-world setting. The goal of the simulation was to identify the better-performing perception module and learning module designs. The selected modules are then transferred to a custom-built mobile robot, conserving the abstract system.

### 4.1. Simulation

We used the CARLA simulation environment for simulating and experimenting with the models. CARLA has a server-mode simulation where simulations can be controlled by Python clients. It has an autopilot mode that is controlled by an internal PID controller, which provides desirable control actions for the current state of the vehicle and was used as the expert in the imitation learning simulation. The Python API in the CARLA environment allows customization of the inbuilt autopilot controls of the simulated vehicle. CARLA provides valuable measurements of the autopilot agent, such as the transformation of the vehicle acceleration, forward speed, intersection of other lanes, and off-road intersection. The autopilot controls comprise the steer, throttle, brake, hand brake, and reverse controls that the AI could take in a particular situation. CARLA provides information about non-player agents, such as vehicles, pedestrians, traffic lights, and speed signs. Further, CARLA is equipped with a rich set of cameras, namely a scene camera, depth camera, semantic segmentation camera, and ray-cast-based LiDAR. During the simulation, we used a CARLA simulation configuration with no non-player agents. This means the simulation environment contained a two-lane road with roadside objects along with the agent vehicle. The speed was set to a constant since generating the control parameter was the emphasis as far as the learning concern was steering.

### 4.2. Physical Implementation in Real World Settings

The system was evaluated on a real-world robot platform evaluated with different tasks. We implemented a robot based on an ROS system built on a custom small-scale robot vehicle platform powered by 6 V differential drive motors with encoders with additional sensor connectable options. We used Arduino ATmega 2560 as a low-level controller to control the motor controller and the NVIDIA Jetson TX2 Development Kit as the main computer running on Ubuntu 16.04 with ROS, OpenCV, and a Jetpack GPU support system. Our platform was capable of supporting Vu8 LiDAR by LeddarTech Inc., MPU91250 IMU sensor by InvenSense Inc., and IR sensors in order to provide expanded observing capability. For our experiment, we used the onboard camera of the TX2 development board and a F710 wireless game pad by Logitech to give expert signals during the training phase. The game pad consisted of two 2-degree of freedom (DoF) joysticks, where one was used to accelerate and break the robot vehicle, while the other was used to steer left and right. Joysticks are different from key presses as they can provide fractional commands. For example, in our implementation, to take a slight right turn, the joystick has to be moved through half of the full range of the joystick toward the right, and to take a sharp right turn, the joystick has to be moved to the far right. For the former, the steering command is +0.5, and for the latter, the command is +1.0. These fractional commands allow the agent to understand the difference between a full turn and a slight turn. The same concept applies to the other joystick that controlled the speed of the robot vehicle, where +1.0 indicates full throttle and +0.5 indicates half-throttle. The driving of the robot vehicle was based on these two control signals.

**Figure 3:**
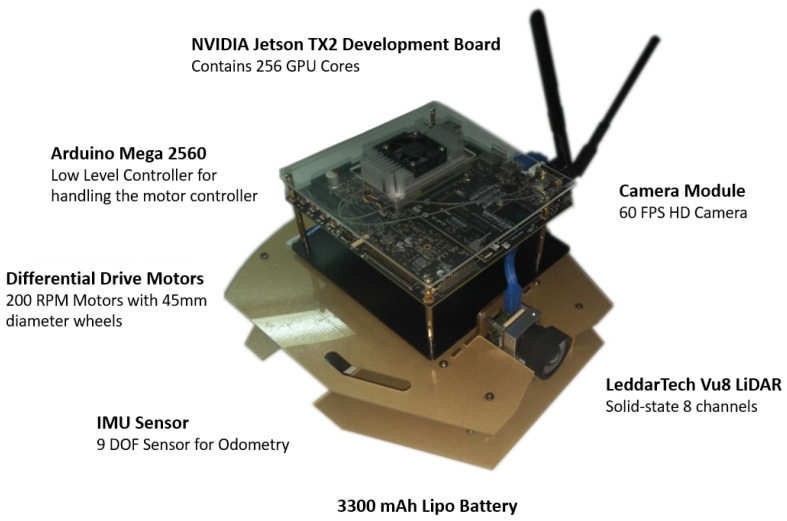
Implemented ROS bot. Although LiDAR is included among the robot sensors, it was not used in this work.

**Figure 4:**
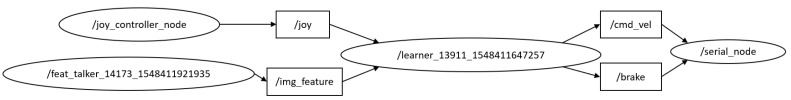
ROS node structure.

**Figure 5:**
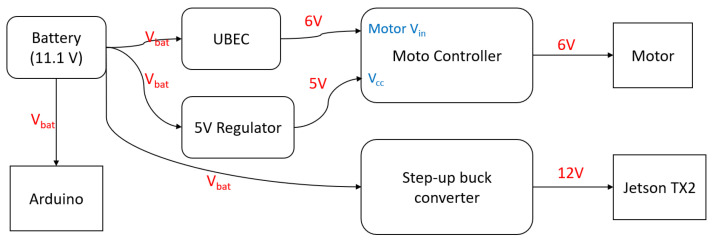
Power diagram of the robot.

All of these systems were integrated (see Figure 3), and the signals were synchronized with ROS nodes for each component (see Figure 4). Communication between the Arduino Mega 2560 and the TX2 board was established through a serial communication protocol. This setup used the *rosserial* library, which bridged the Arduino platform with the ROS ecosystem, ensuring a seamless and standardized interface for data exchange and command relay between the microcontroller and the ROS-based system. To power the robot, an 11.1 V Lipo battery was used with battery elimination for different voltage levels required (see Figure 5).

## 5. Experiments & Results

In this section, we present the results obtained when experimenting with the different alternative models that constituted the imitation learning system.

### 5.1. Performance Evaluation

A common error metric called the average error per frame (AEF) was defined for each trained policy to reflect the performance and the convergence of the training iterations for the simulation and the real-world experiments. The formulae for the simulation and the real-world experiments differ, as the ability to measure some physical parameters are different in the two settings, but both formulae are motivated to capture the deviation from the expert’s and training agent’s trajectories. The trajectory is the position of the robot vehicle at a given time. The expert’s trajectory is the trajectory of the robot vehicle if it is controlled by the expert. The AEF used in the simulation setting is given by the following expression.

(1) $$\mathrm{AEF}=\frac{1}{f}\sum_{i=1}^{f}\left(x_{1}^{i}+x_{2}^{i}+x_{3}^{i}\right)$$

Here, $x_{1}^{i}$, $x_{2}^{i}$, and $x_{3}^{i}$ are parameters depicting a collision, the intersection of the vehicle with the other lane, and the intersection of the vehicle with area off the road respectively. *f* is the total number of frames for a given simulation episode. $x_{1}^{i}\in \left\{0,1\right\}$ is a Boolean value depicting whether there was a collision in the $i^{th}$ frame, where 0 denotes no collision and 1 denotes a collision. $x_{2}^{i},x_{3}^{i}\in \left[0,1\right]$ depicts the proportion of the vehicle intersecting the other lane and the off-road area in the $i^{th}$ frame. Here, 0 denotes no intersection, and 1 denotes full intersection. Due to the dimensions of the road and the vehicle, for a given frame, $x_{2}^{i}+x_{3}^{i}\leq 1$. As evident from Equation (1), AEF is the average sum of $x_{1}^{i},x_{2}^{i}$ and $x_{3}^{i}$, which gives AEF≤2.

For the real-world implementation, AEF is defined as the deviation of the agent’s trajectory to the expert as in Equation (2). This is due to the inavailability of the exact coordinates of the robot vehicle in the real-world arena.

(2) $$\mathrm{AEF}=\frac{1}{f}\sum_{i=1}^{f}\left|{\mathrm{steer}}_{\mathrm{agent}}^{i}-{\mathrm{steer}}_{\mathrm{expert}}^{i}\right|$$

Here, ${\mathrm{steer}}_{\mathrm{agent}}^{i},{\mathrm{steer}}_{\mathrm{expert}}^{i}\in \left[-1,1\right]$ represent the agent control output and expert control output, respectively, for a given frame, where −1 denotes a hard leftward turn and 1 denotes a hard rightward turn. *f* is the number of frames per episode. AEF is the average absolute difference between the expert and agent action decision per frame. Hence, AEF≤2. The intuition behind AEF is to capture the deviation of the training agent from the expert. If the training agent generates steering angles that are largely different from the expert, Equation (2) will evaluate to a higher value, and if not, to a lower value.

When training an agent to perform a specific task, it is expected that the AEF metric for each policy iteration in a training session will have some relationship because we are optimizing the same policy with more data as per Algorithm 1. In other words, intuitively, one can expect the next adjacent policies to generate lower AEF values provided the learning architecture is proper, but practically, this is not the case. The AEF values generated over a training session tend to be noisy, as a small change in the training episode can cause the agent to travel to unexplored territory. For example, if one had a very low AEF in the previous training episode, the next episode could generate a high AEF due to a small directional or positional change in the starting position. Therefore, the initial training iterations were expected to be chaotic and unpredictable, but as more iterations progress, this was expected to decrease. This was the reason for the high-noise nature of the AEF graphs in the experiments.

### 5.2. Simulation

**Figure 6:**
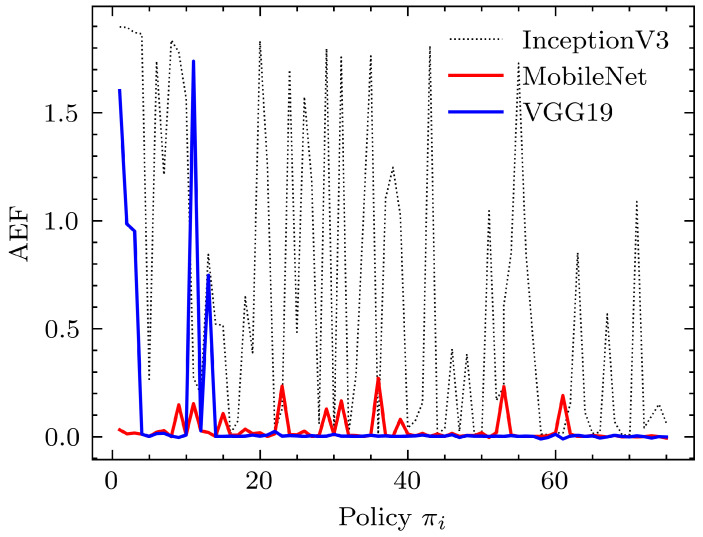
Performance comparison of selected CNN architectures as the perception module. The learning module was kept the same and used a single dense layer followed by a tanh activation neural network (β${}_{0}$=0.7). The reason for the noisy nature of the graphs is explained in Section 5.1.

Simulations were carried out mainly to obtain insights into how to design the perception and the learning modules. For the perception module, 3 mainstream deep learning CNN feature extraction methods, MobileNet, VGG19, and InceptionV3, were used. In all these experiments, the last classification layer of the CNNs was removed to obtain a hypervector that represents the scene in each of the frames. Figure 6 shows how AEF behaves for each of the CNN architectures.

A better perception module architecture was expected to drop the AEF rapidly. As seen in Figure 6, performance with the InceptionV3 [51] network is irregular and often yields higher AEF values. Comparatively, MobileNet [47] and VGG19 [52] seem to perform well, and their performance is similar. It should be noted that we used single-layer dense architectures with 1024, 512, and 2048 parameters (plus a bias parameter) for MobileNet, VGG19, and InceptionV3, respectively, to accommodate their respective feature outputs. Despite the performance similarity, the VGG19 network has 20,024,384 parameters compared to 3,228,864 parameters in MobileNet. (These numbers correspond to the parameters in the respective pre-built Keras [53] models, taking only the convolutional filters). This translates to less memory and computation intensiveness of MobileNet compared to VGG19 while delivering similar performance with regard to the AEF and making the perception module lightweight.

**Figure 7:**
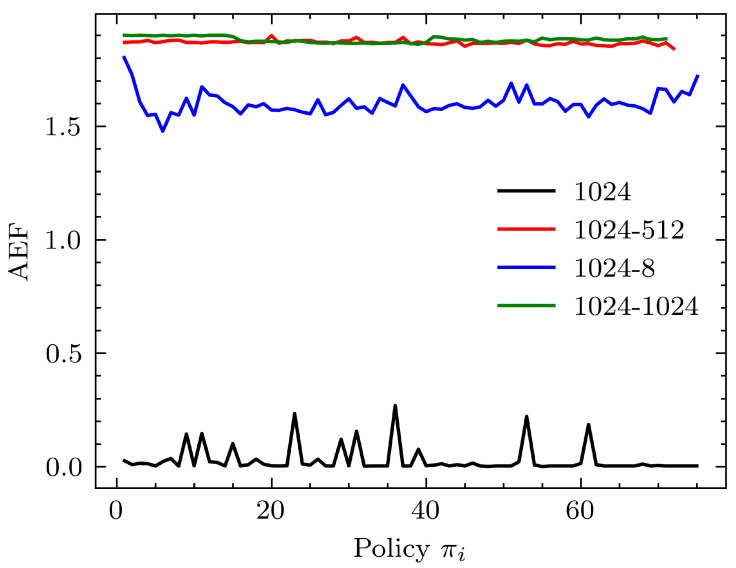
Performance comparison of selected dense layer architectures as the learning module. The tanh function was the activation used at the output for all dense layer architectures. The perception module used was MobileNet followed by an average pooling layer (β${}_{0}$=0.75).

For the learning module, several dense layer architectures were tested and compared. As seen in Figure 7, we tested 1024, 1024-8, 1024–512 and 1024–1024 dense layer configurations. The configuration is in the form (1st layer parameters)–(2nd layer parameters (if present)), omitting the bias parameter. Testing was limited to 2 dense layers since the emphasis was on lightweight models. We used 1024 parameters in the 1st layer in all test cases, as the perception module output was 1024 features with MobileNet. The experiments demonstrate that the performance of the learning system with regard to AEF is better for the single-layer 1024 configuration. A slower rate of responsibility shift may be needed for larger networks for better policy evolution, as the data needed for training may be high for larger networks.

**Figure 8:**
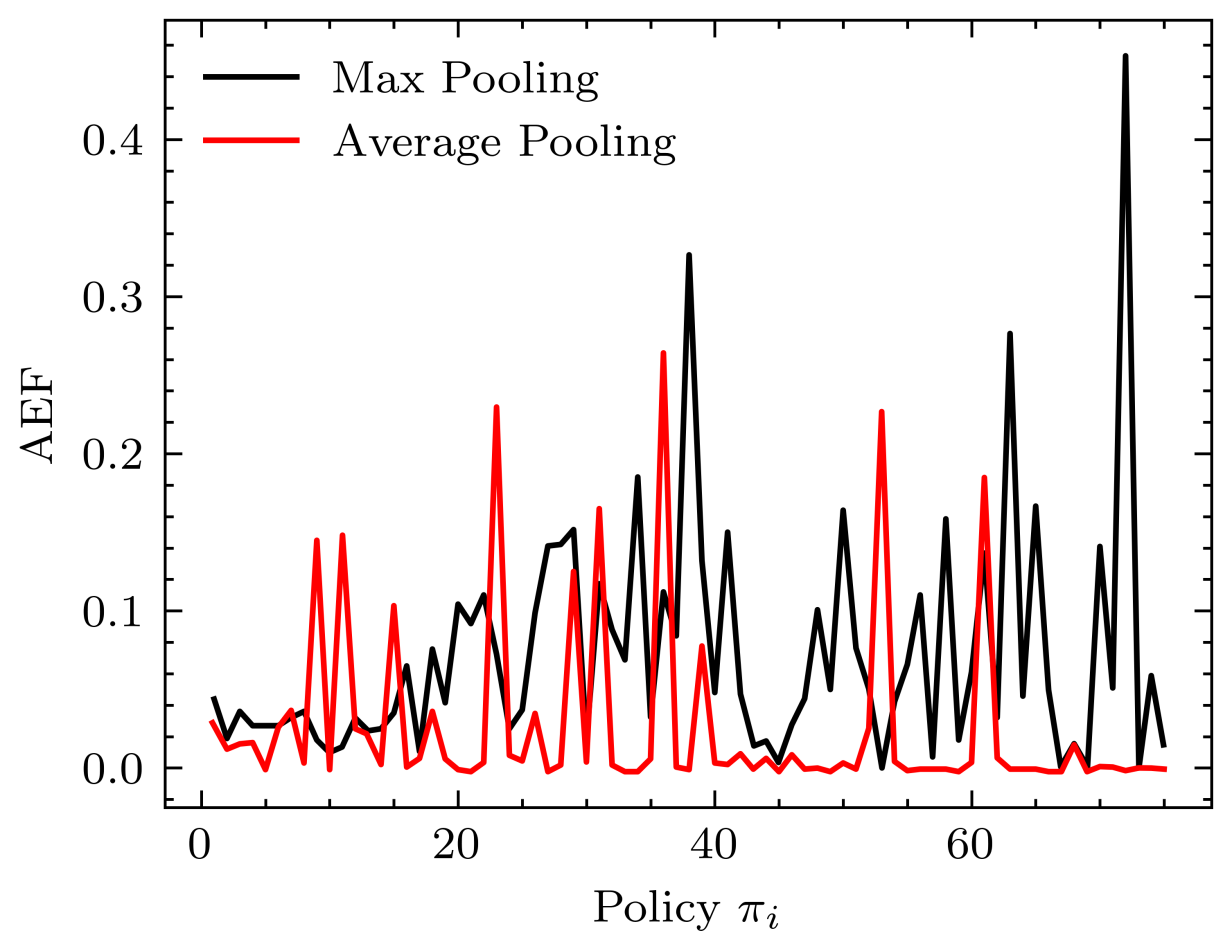
Performance comparison of selected pooling layers to be used after the perception module. The learning module used was a single dense layer of 1024 units with tanh activation at the output. The perception module used was MobileNet (β${}_{0}$=0.7).

Comparing the performance of the learning system with different pooling layer types to extract 1024 features from the convolutional filter outputs was carried out using average and max-pooling layers. Figure 8 shows the performance.

The results suggest that the performance of the average pooling layer is slightly better than that of the max pooling layer with respect to the AEF. When using average pooling, the results were stable in terms of performance compared to the varied noisy performance when using max pooling. This may suggest that when extracting features from the filters of the CNN, focusing on overall average features rather than sharp prominent features is desirable in the context of predicting the steering direction for a given image frame using a single dense layer network.

**Figure 9:**
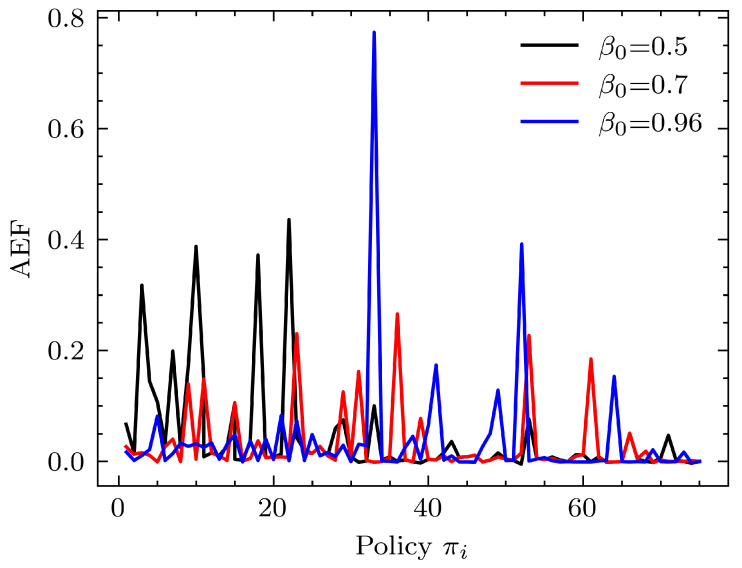
Performance comparison for different β${}_{0}$. The learning module used was a single dense layer of 1024 units with tanh activation at the output. The perception module used was MobileNet.

In the learning algorithm, $\beta_{0}$ determines how the responsibility of control is transferred from the expert to the agent as the training iterations increase. Simulations were carried out under different initial $\beta_{0}$ values using MoibileNet as the perception module and a 1024 linear regressor as the learning module. Figure 9 shows their performance results.

From Figure 9, we can observe that performance improvement happens for every beta value tested ($\beta_{0}$ = 0.5, 0.7, 0.95) as the number of training episodes increase. However, AEF values for $\beta_{0}$ = 0.5, 0.95 are slightly higher than that for $\beta_{0}$ = 0.7. Thus it can be seen that operating at $\beta_{0}$ values that are neither too low (>0.5) nor too high (>0.95) is desirable for good performance evolution. This makes physical sense since when operating at $\beta_{0}<0.5$ values, the major part of the control of the robot is with the agent, which is closer to a reinforcement learning problem formulation, and $\beta_{0}>0.95$ is closer to a generic supervised learning problem.

As a final remark, it can be said that having a $\beta_{0}$ value high enough is essential so that the expert has enough control early on to navigate the robot in the states of high importance rather than demonstrating how to navigate in states of lesser importance. Furthermore, these results show the performance of the learning system we are presenting with different responsibility shifts from expert to agent. The spikes between low AEF values account for the phases where the agent is exploring previously unseen states due to the responsibility shift. In Figure 9, for $\beta_{0}=0.5$, significant and frequent early spikes are seen for the policy evolution, but low spikes are seen towards the end. For $\beta_{0}=0.95$, low spikes are seen towards the beginning, and significant spikes are seen towards the end. These observations can be explained as a lack of responsibility taken by the expert in guiding the agent initially and too much responsibility taken by the expert in guiding the agent initially, respectively. $\beta_{0}=0.7$ is a middle ground between these two extreme ends.

In summary, for the real-world implementation, we used $\beta_{0}=0.7$ for the modified DAgger algorithm, the transfer-learned convolutional layers of MobileNet with average pooling to obtain 1024 feature-extracted hypervectors, and two linear regressors of 1024 parameters (without bias) with tanh activation for steering and sigmoid activation for the safety control policy.

### 5.3. Physical Implementation

**Figure 10:**
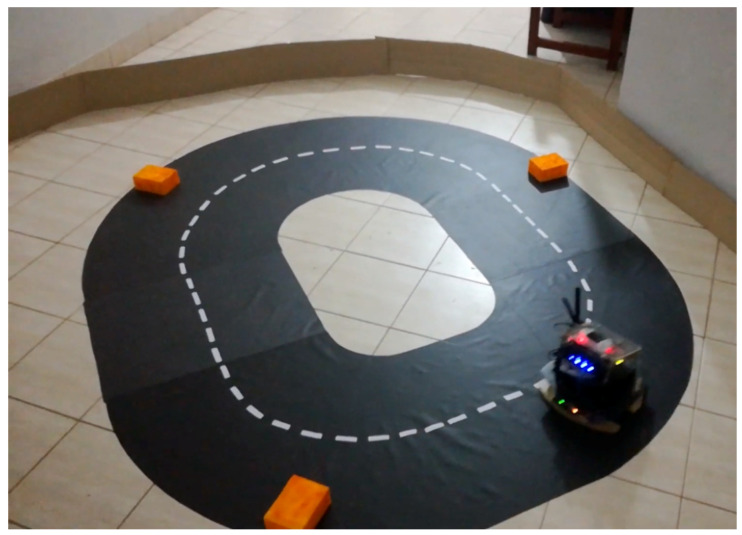
Real-world task demonstration environment for an obstacle avoidance task in a two-lane road (Experiment 4). The agent was expected to navigate on the right side of the road, and once an obstacle is near, to avoid it by traveling to the other lane partially and returning to the correct lane.

Performance evaluation for the real-world implementation of the imitation learning system was carried out using several tasks performed in an artificial environment setting. This environment setting is shown in Figure 10. In order to evaluate the performance of the system, 4 different navigational experiments were carried out:

1. Navigating on a two-lane road on the right side only;
2. Braking at the wall of the arena;
3. Traveling around a static obstacle;
4. Navigating on a two-lane road while overtaking static obstacles by partially traveling to the other lane and returning.

The experiments were numbered according to their order of complexity, and the final experiment was a combination of all others, imitating a real-world driving scenario. It should be noted that we do not provide performance evaluations on Experiments (1)–(3), as the final experiment is a combination of them. We do not evaluate braking due to the lack of a reliable metric and due to the complexity of generating one. Therefore, the iterations where the robot became unresponsive and braked at the wall were omitted from evaluating the performance in Experiment (4).

## 6. Discussion

**Figure 11:**
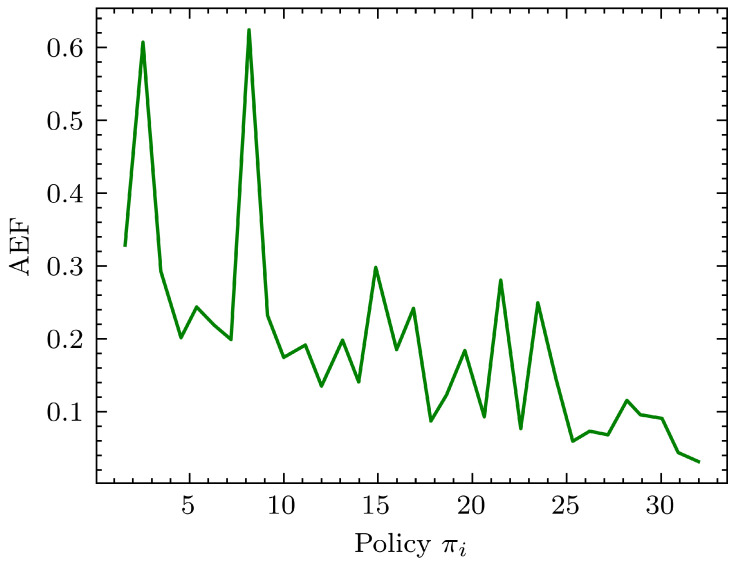
Performance evaluation of the agent for obstacle avoidance in a double-lane road task using a trained agent. We observed that the agent converges fast and performs satisfactorily after 10–15 training iterations. Policies after 25–30 training iterations showed signs of over-fitting, where the robot vehicle took unexpected turns.

Here, we present the performance evaluation results of obstacle avoidance in a double-lane road task. In this task, the robot needed to travel in the right lane as long as there was no obstacle. If it met with an obstacle, it was intended to avoid the obstacle by switching to the left lane and returning to the right lane as soon it passed the obstacle. The agent was trained by a human expert controlling the robot with a Logitec-F710 wireless game pad remotely. The performance evaluation results are shown in Figure 11.

It should be noted that for the AEF calculation, we used a well-performing pre-trained agent as the expert, rather than a human expert. The reason for this is for comparison purposes for a given frame. Especially towards the latter part of the training when the human has lesser control over the robot, the control input given by the human expert is noisy since they tended to try to take over the control subconsciously, although the imitation learning algorithms gradually passed the control to the agent. As a result, although it can be observed that learning agents started to maneuver in a desirable manner, AEF shows no clear performance improvement. An artificial agent, on the other hand, provides consistent control outputs irrespective of its control over the robot’s movement.

As a safety maneuver, another linear regressor network independent of the steering network was added to the learning module to teach the agent braking. This was added due to the occasional unpredictability of the agent convergence and to avoid damaging the robot. The agent was trained by a human expert to stop the throttle whenever it reached the wall in the arena. It should be noted that we do not provide performance evaluation on braking tasks due to the lack of reliability and complexity of generating such a performance evaluation other than as visual observations.

**Figure 12:**
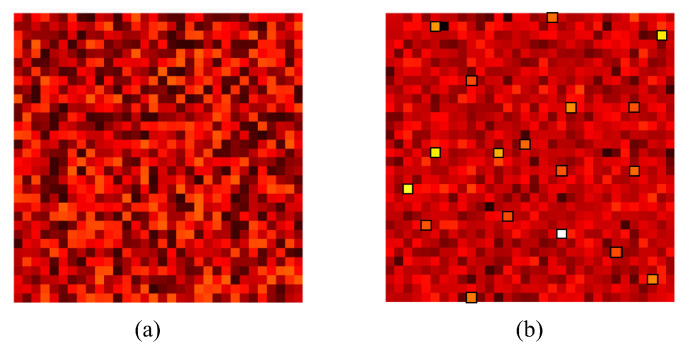
Learning by the agent to focus on key image features for the steering task. (**a**) Randomly initialized weights of the learning module steering network. (**b**) Learning module after learning to steer.

**Figure 13:**
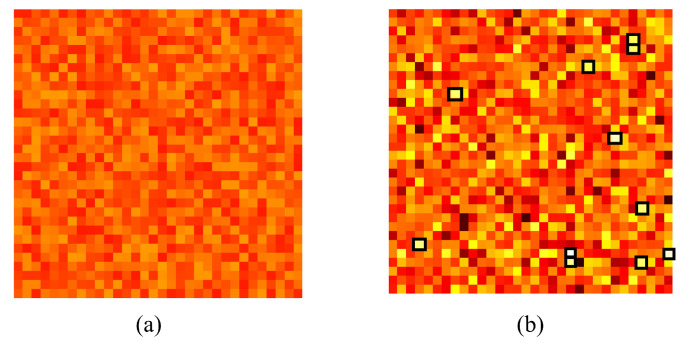
Learning to focus on key image features for the braking task. (**a**) Randomly initialized weights of the learning module braking network. (**b**) Learning module after learning braking.

**Figure 14:**
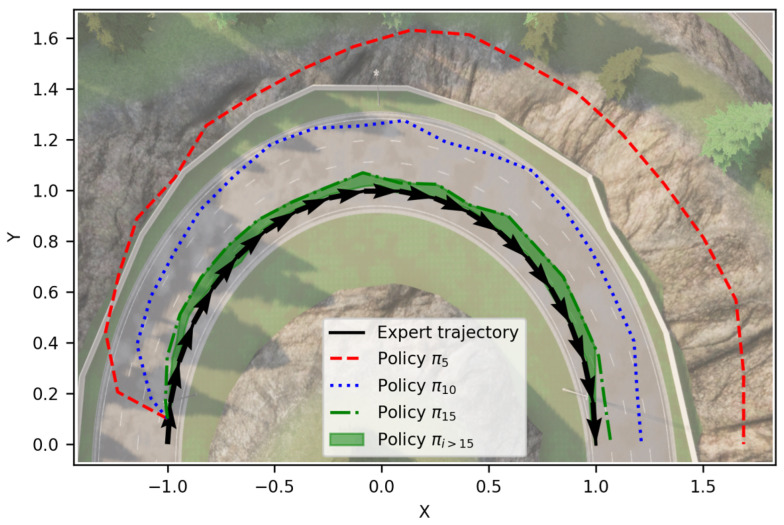
Trajectories of agent policies during training demonstrated in CARLA Map 6 while taking a 180° turn. The expert trajectory used here is the CARLA Autopilot (shown in black). Initial training policies tend to deviate largely from the expert (shown in red and blue). As the training iterations increase, the training agent starts performing much more similarly to the expert (shown in green). Around Policy $\pi_{15}$, the agent starts showing close behavior to the expert. The X and Y axes are centered and scaled down for visualization purpose.

Analysis of the weights of the linear regressor of the learning module reveals that a specific set of weights have been altered drastically compared to others in both the steering and the braking networks. Figure 12 and Figure 13 show the randomly initiated and after-training learning module for the steering and braking tasks, respectively. Each square pixel in the matrix corresponds to a learning module weight given to a feature output from the perception module. The set of 1024 weights (without bias) in the learning module was arranged to form a 32 × 32 pixel matrix for convenience of presentation. The lighter the color of the pixel, the higher the numerical value; thus, the higher the attention to the corresponding feature. Also, it should be noted that learning module pairs for steering and braking are not comparable, as the weight pixels are assigned colors according to the minimum and maximum values attained by the weights in that specific learning task. Thus, the emphasis is on the relative attention to different features for a given task. In Figure 14, we illustrate how the proposed methodology converges to the expert policy in a simulated experiment of a 180-degree turn in the CARLA map 6 simulation environment, where as the training iterations increase, the training agent starts performing similar to the human expert.

### 6.1. Simulation-to-Reality Challenges

In this section, we discuss the challenges we faced during the transfer of the model from our simulation to the real world. One of the main challenges faced during real-world training is the dependency of model performance on ambient lighting conditions. A slight change in the lighting affected the performance of the driving task severely. In order to tackle this, we surrounded the arena with a wall so that the camera would capture important features in the road such as lane-dividers and obstacles while having a uniform background. This improved the sudden variability in performance during early experiments.

Although we trained a safety control policy to control the throttle of the robot vehicle, occasionally, it traveled far away from the road at high speeds. Since the agent has more control over steering at the latter stages of training, it is hard to stop it before it damages itself. Therefore, we added a start/stop switch to cut off power to the motor controller from the Arduino as a safety maneuver. This prevented unexpected damage to the robot hardware and to the arena.

The metrics used to evaluate simulation performance in CARLA were obsolete in the real world. Unlike in a simulation, there is no way to access state parameters reliably in the real world except as estimations. Therefore, designing an evaluation metric was a challenge. In addition to that, the human expert control was unexpectedly noisy. This is especially due to the transfer of control to the agent as training episodes increased. The human expert started to feel the commands passed to the robot did not have much of an effect on the robot anymore as the training proceeded. Therefore, unintentional control signals were expected from the human, making the expert data noisy. Low-pass filtering of the expert signal significantly increased the performance of the system. These filtered expert commands were used in the real-world task evaluation metric (2).

### 6.2. Limitations

As explained in Section 6.1, the proposed system reacts to changes in ambient lighting. Since we did not train the feature extraction convolutional deep neural network during imitation learning, this cannot be directly addressed. Therefore, changes in the environment affect the performance of the system, as the state estimation is not robust to noise from the environment. To overcome this, transfer learning on the CNN can be carried out before operating in a new environment. This has to be carried out in an offline setting by collecting the camera images from the robot vehicle and training the CNN for a few episodes using a more resourceful computer.

During training, it was noticed that the learning module could not maintain plausible performance for several tasks at once. For example, once the agent is trained to travel on the right side of the road with obstacle overtaking, it is hard for it to train the same way for the other lane without losing performance on the prior task. The agent is only capable of holding the last trained task. This catastrophic forgetting is a result of the small number of trainable parameters in the learning module.

## 7. Conclusions

In this article, we have proposed a lightweight, quickly convergent direct policy learning approach for resource-constrained mobile robot autonomous navigation. We presented a structured system that mainly consists of a perception module, which filters sensory inputs, and a learning module, which can learn to imitate some experts by matching states with actions taken by the expert. Specifically, this system acts as a deep regression model with a trainable dense layer component. We evaluated this approach on both simulated and real-world implementations in resource-constrained settings. We compared the lightweight components with heavyweight counterparts and demonstrated a performance improvement for baseline tasks. We also demonstrated fast convergence in the obstacle avoidance task on a two-lane passing simulation and high noise in expert control for accurate performance evaluation.

We have planned several activities as future work related to this project. Firstly, we plan to compare our results with related hardware-dependent DPL methods, which were excluded from the current phase due to the high overheads of designing the hardware system to support multiple different learning algorithms. Next, we intend to provide the agent with temporal memory by adding past state vectors as inputs to the learning module, as well as adding other sensors in different directions other than the RGB camera at the front. This would provide a more generalized version of the approach proposed in this paper. Each additional sensory input may or may not be processed and filtered in dedicated regions of the perception module, but an aggregated state representation would be provided for the learning module for the purpose of learning. Here, we address the limitation of the robot neither having temporal memory nor a wide range of sensation of the environment, due to which the robot is unable to respond correctly in some given tasks. In order to explore the generalization capability of the proposed methodology, it is necessary for it to be benchmarked on a wide range of real-world tasks, which we will also complete as future work.
